# Chemical Recycling of Poly(ethylene terephthalate) to Functional Glycolysates: Overcoming Phase Instability and Secondary Crystallization

**DOI:** 10.3390/polym18161961

**Published:** 2026-08-11

**Authors:** Marek Lewandowski, Przemysław Kosobucki, Jacek Stuczyński

**Affiliations:** 1 Purinova Sp. z o.o., Ul. Fordońska 74, 85-719 Bydgoszcz, Poland; j.stuczynski@purinova.com; 2 Doctoral School, Bydgoszcz University of Science and Technology, Al. Prof. S. Kaliskiego 7, 85-796 Bydgoszcz, Poland; 3 Faculty of Chemical Technology and Engineering, Bydgoszcz University of Science and Technology, Ul. Seminaryjna 3, 85-326 Bydgoszcz, Poland; p.kosobucki@pbs.edu.pl

**Keywords:** poly(ethylene terephthalate), chemical recycling, glycolysis, stability, depolymerization

## Abstract

Poly(ethylene terephthalate) (PET) waste management faces challenges as mechanical recycling limitations become apparent under strict EU regulations. This review critically evaluates PET glycolysis as a vital chemical recycling method, focusing on overcoming barriers to industrial implementation. While systematizing knowledge on reaction mechanisms and parameters, a significant research gap is identified: the necessity for utilizing a high initial mass fraction of waste PET in the reaction feed. Specifically, exceeding a critical concentration of PET-derived oligomers in the resulting glycolysis reaction mixture (typically when the initial waste PET input is above 40% by mass) inevitably triggers phase instability and secondary crystallization during storage. This instability at high concentrations is fundamentally driven by the altered oligomer molecular-weight distribution and the thermodynamic supersaturation of rigid aromatic segments upon cooling. Traditional laboratory approaches using a high excess of glycolyzing agent fail to meet industrial stability demands for subsequent polyester polyol synthesis. Currently, preventing crystallization relies on costly branched glycols or modifiers to disrupt molecular symmetry. This article highlights the urgent need for alternative, additive-free methods to achieve phase stability, such as the elimination of released ethylene glycol from the reaction environment. By addressing shortcomings in glycolysate shelf-life studies, this review charts innovative directions for developing technologies that convert high concentrations of waste PET into phase-stable glycolysates.

## 1. Poly(ethylene terephthalate)

**Figure 1:**
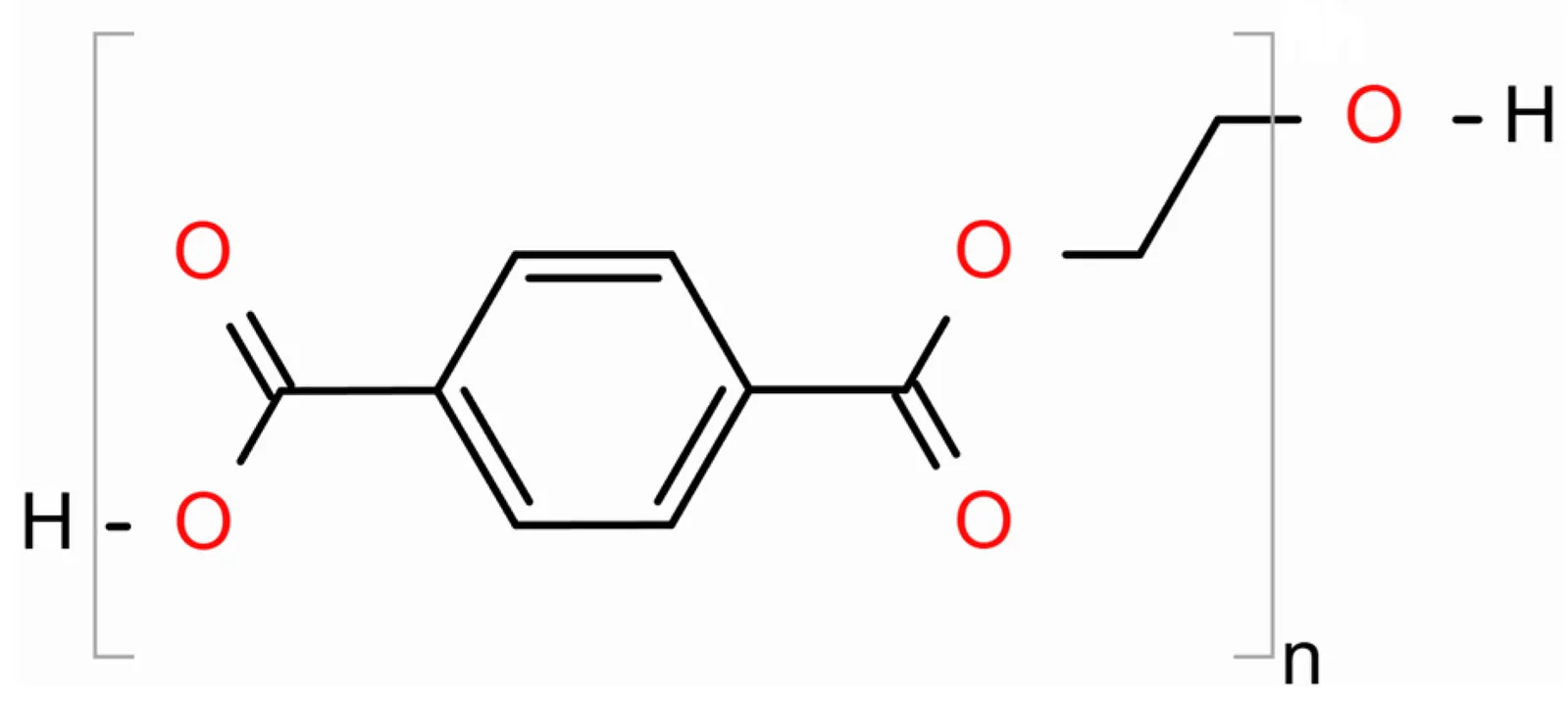
Chemical structure of poly(ethylene terephthalate).

Poly(ethylene terephthalate) (PET) is one of the most important and widely used thermoplastic polymers in the world [1,2]. Global production of PET currently exceeds 85 million tons per year, with the European market consuming approximately 15% of the global volume [3,4,5]. PET is a semi-crystalline polymer whose chemical structure consists of aromatic rings separated by short monoethylene glycol (MEG) segments, as shown in Figure 1.

The high regularity of the polymer chain and the presence of rigid, symmetrical aromatic rings determine its excellent barrier and mechanical properties, as well as high thermal resistance [6]. The global market is dominated by the textile sector, which accounts for over 60% of total PET production, whereas in Europe, the packaging market prevails, accounting for as much as 96% of the European consumption of this polymer [4,7]. The pharmaceutical industry (medical packaging) [8] and the construction sector (insulation, membranes, sound-absorbing barriers) [9] also remain significant areas of PET application.

**Figure 2:**
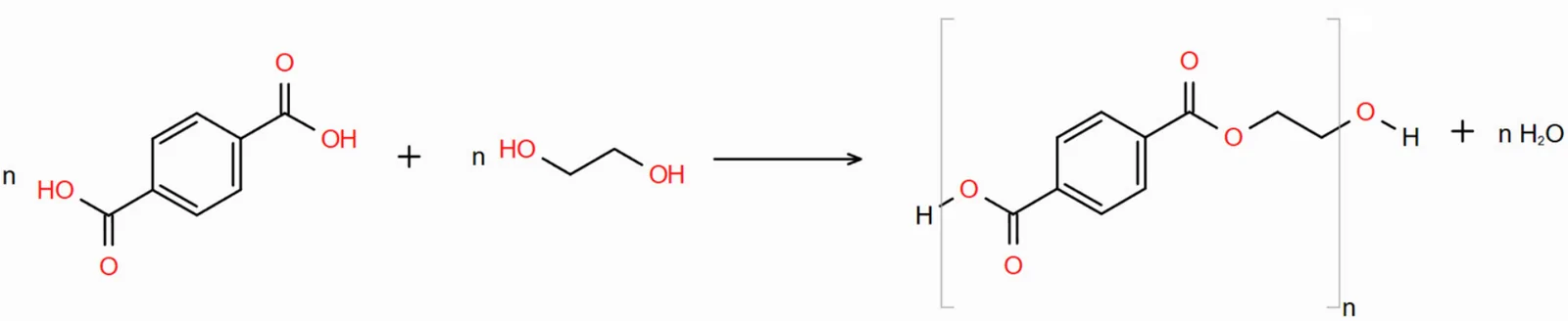
Reaction scheme of esterification of PTA with MEG.

The industrial-scale production of PET is based on two main technologies. Currently, the dominant method, covering over 90% of global production, is a process involving the direct esterification of purified terephthalic acid (PTA) with MEG, followed by the polycondensation of the obtained prepolymers [10]. The by-product of this process is water. The reaction scheme is shown in Figure 2.

**Figure 3:**
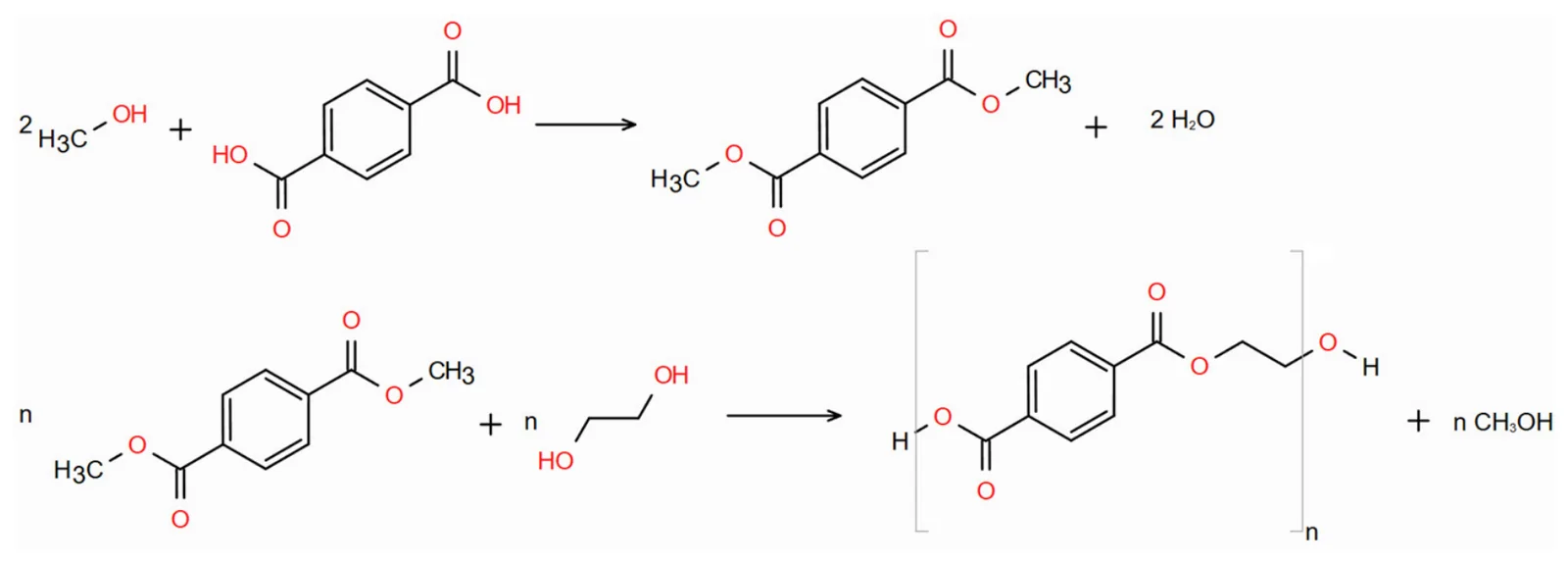
Reaction scheme of the esterification of PTA with methanol, followed by the transesterification of DMT with MEG.

An alternative technology is the transesterification of dimethyl terephthalate (DMT) with MEG. The fundamental difference compared to the PTA method is that when using DMT, the by-product is methanol, which, due to its toxicity and flammability, requires complex recovery and management technologies [11]. The reaction scheme of the DMT process is shown in Figure 3.

## 2. PET Recycling

### 2.1. Scale of Production and Waste Generated

**Figure 4:**
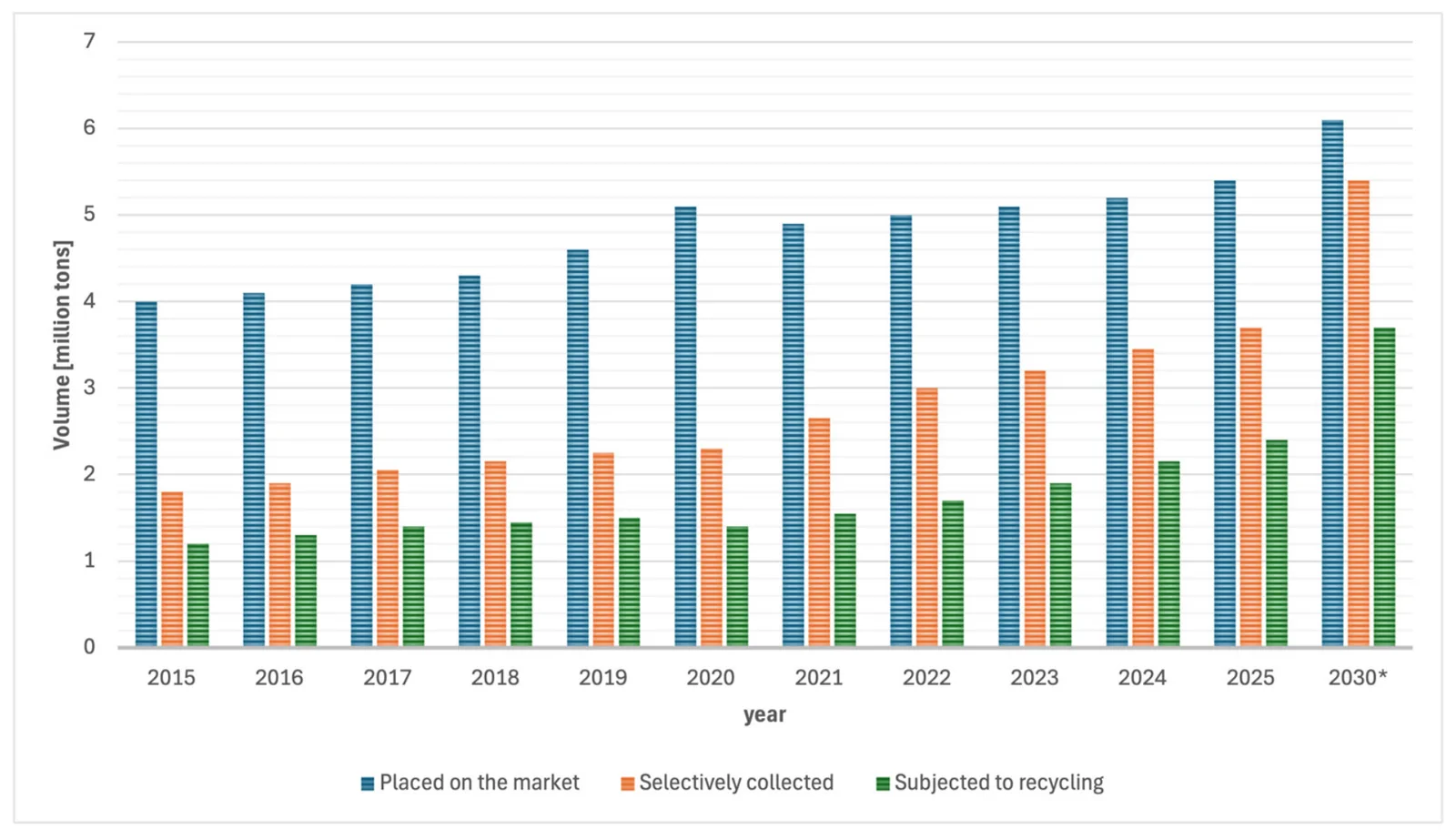
PET packaging volume placed on the market and the corresponding stream collected and subjected to recycling in EU countries [18]. (*—forecast for 2030).

The environmental degradation time of PET is estimated to be over 400–500 years [12,13]. The average service life of PET packaging products is estimated to be less than 6 months [14], which, combined with the large scale of production, contributes to a growing problem regarding the management of generated waste, including textile waste. It is estimated that approximately 4.3 million tons of PET waste are generated annually in the EU countries [15]. Forecasts indicate that if the current economic model is maintained, volume may double by 2040, reaching a level of 7 to 8 million tons in the EU [16]. The historical trends and future projections for the volumes of PET packaging placed on the European market, alongside the collected and recycled fractions, are presented in Figure 4. The environmental persistence of PET waste is primarily driven by the polymer’s high durability and poor biodegradability, presenting a major challenge in regions with inadequate waste management infrastructure [17]. Landfilling PET waste is the least desirable solution for both environmental and economic reasons, as it represents an irretrievable loss of a valuable raw material.

**Figure 5:**
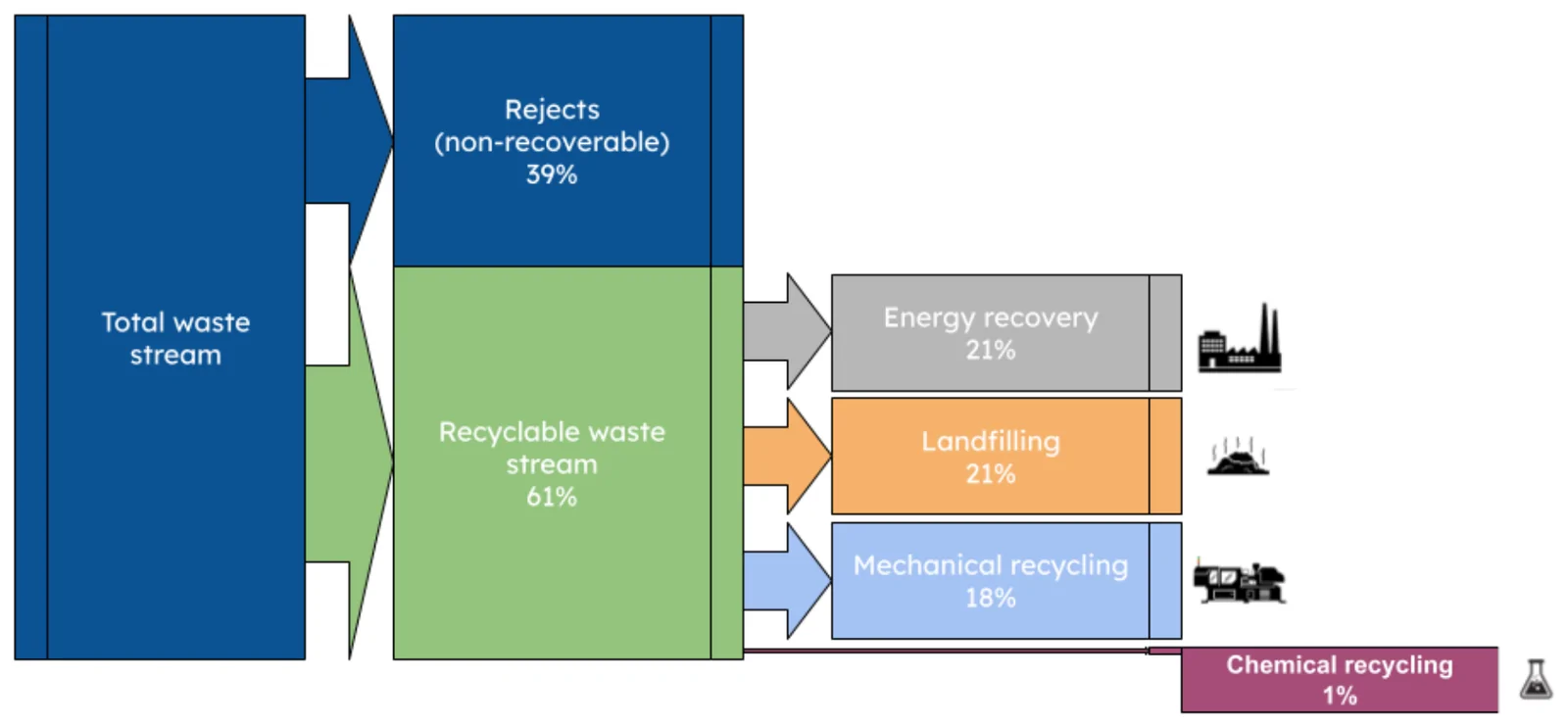
Global estimated mass flow and end-of-life pathways for total PET waste (including packaging, textiles, and industrial applications) [16].

The level of selective collection of PET packaging waste in Europe in 2022 reached 60% (an increase from 45% in 2020) [19], making PET packaging one of the leading recycled polymer streams regarding recycling volume (approximately 3.7–3.8 million tons annually), second only to polypropylene (PP), polyethylene (PE) and poly(vinyl chloride) (PVC) [20]. However, this high collection efficiency is specific to European packaging. In contrast, the global mass flow of total PET waste exhibits much lower mechanical recycling rates and substantial losses to landfills or energy recovery. Further end-of-life pathways for this stream, categorized by the recycling technologies applied, are presented in Figure 5.

### 2.2. Legislation and Recycling Limitations

**Figure 6:**
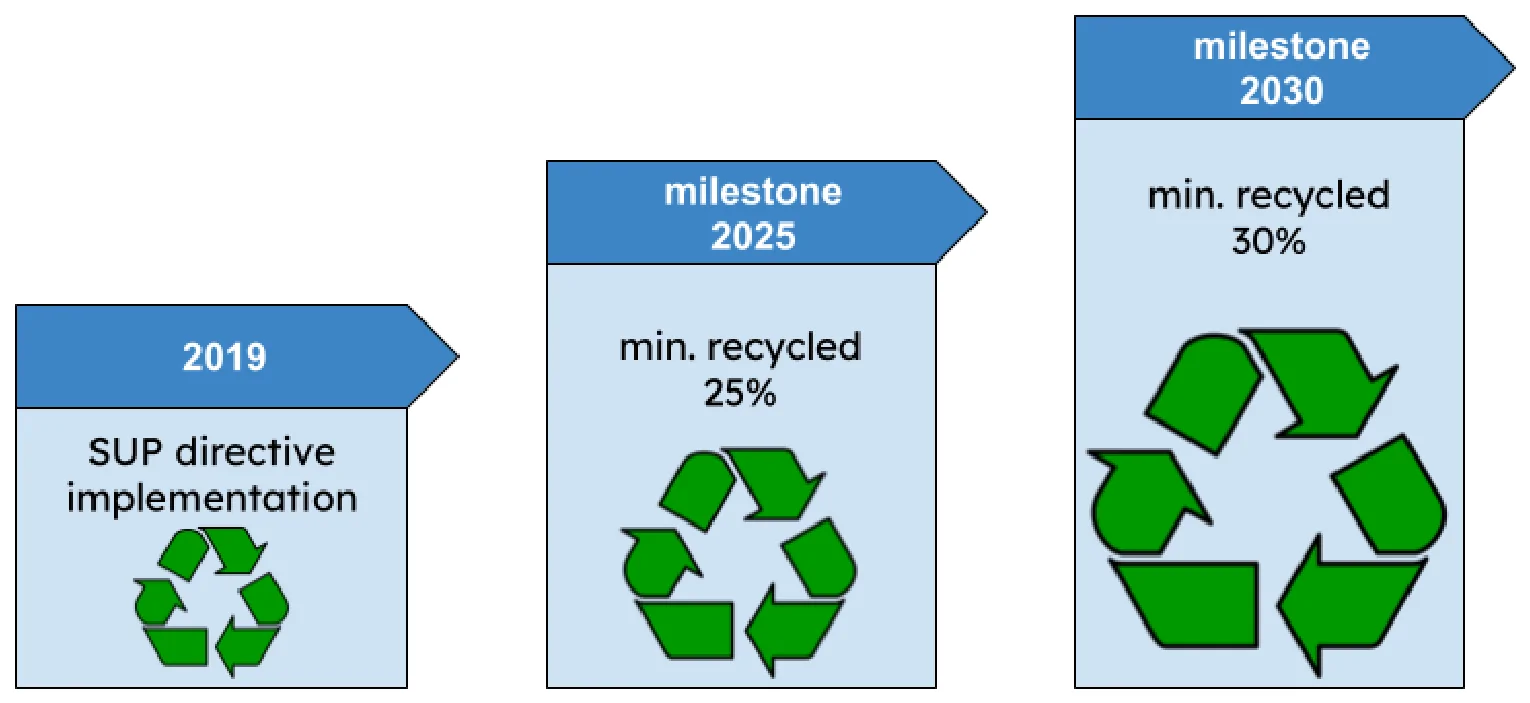
Single-Use-Plastics Directive implementation and recycled content requirements for PET beverage bottles.

The exponentially growing amounts of PET waste have forced decisive actions from legislative authorities at the EU level. A breakthrough moment was the introduction of the Single-Use Plastics (SUP) Directive in 2019 [21], which restricted the market placement of single-use PET products and set rigorous guidelines for the circular economy. This document, acting as an integral part of the European Green Deal, establishes milestones regarding the recycled content specifically in PET beverage bottles [22]. It will be mandatory to include 25% by 2025 and 30% by 2030, as shown in Figure 6 [21,23]. While the SUP Directive focuses on packaging, the substantial polyester textile waste stream is expected to be addressed shortly by the upcoming Ecodesign for Sustainable Products Regulation (ESPR), which targets durability, recyclability, and recycled content requirements for textiles.

**Figure 7:**
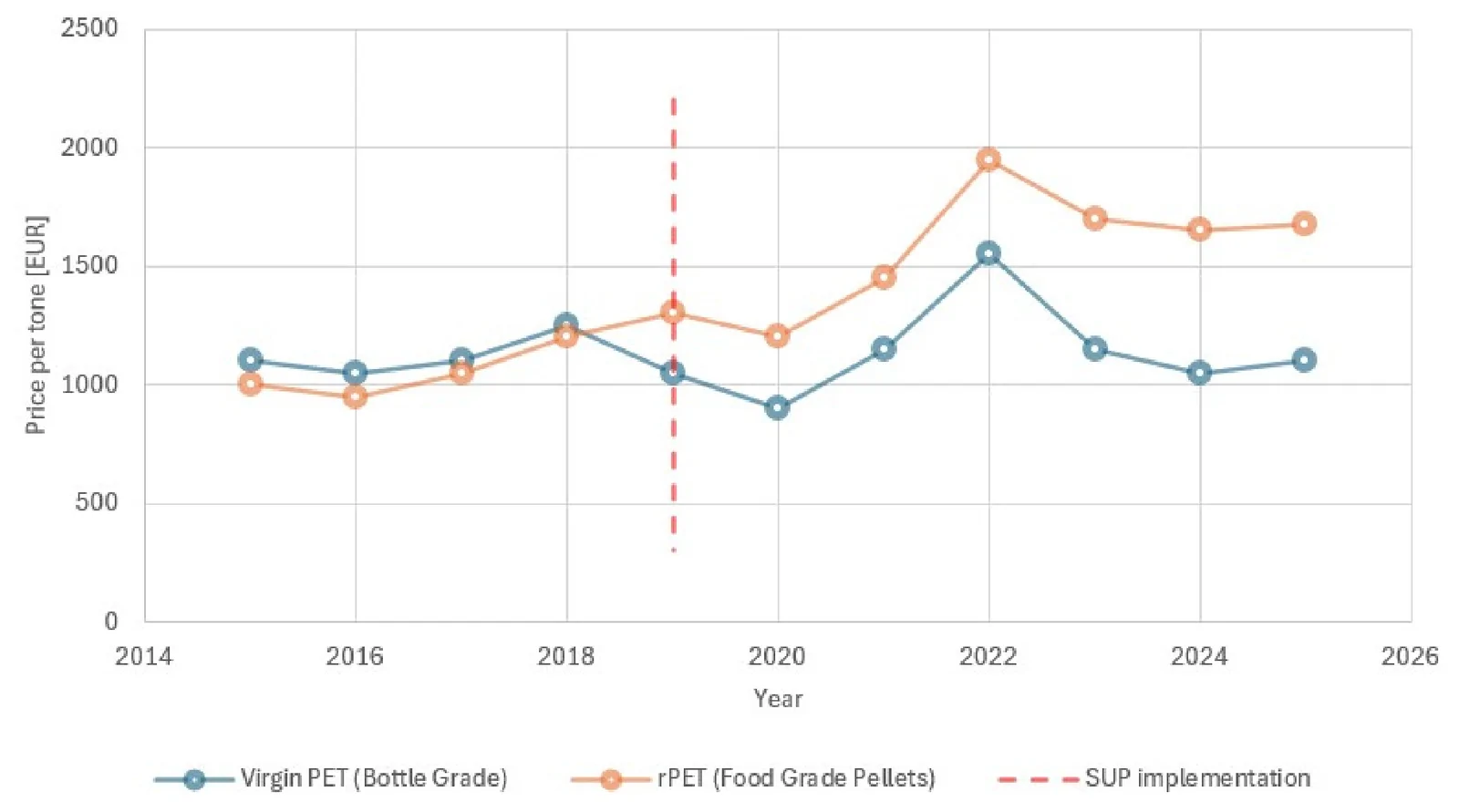
Comparison of virgin PET and rPET market prices in relation to the Single-Use-Plastics Directive implementation.

The introduced regulations clearly affect the market prices of virgin PET and recycled PET (rPET) in European markets. The implementation date of the SUP Directive coincides with a trend reversal, where the price of rPET permanently exceeded the price of the petrochemical raw material, with this price gap systematically growing. This correlation is illustrated in Figure 7.

The implementation of these regulations poses a severe technological and logistical challenge. Every processing cycle, comprising collection, sorting, cleaning, and reprocessing, contributes to the thermal, oxidative, and mechanical degradation of the polymer chain, resulting in a progressive deterioration of its physicochemical properties [24]. Studies indicate that after approximately 5–6 cycles of the same polymer, the degradation is so advanced that the material ceases to meet the required mechanical and processing parameters, permanently disqualifying it from further circulation [25,26].

Despite high collection rates, currently, an average of only 35–40% of collected PET bottles in EU countries are successfully subjected to closed-loop recycling (bottle-to-bottle) [27]. It is important to note, however, that this European average conceals significant regional disparities. For instance, countries with well-established deposit-return systems, such as Germany, achieve collection rates of approximately 95%, with over 50% of the material successfully routed back into closed-loop bottle applications. Conversely, in regions lacking such infrastructure, a substantial portion of the remaining stream still ends up in landfills or is directed to energy recovery. In the face of the adopted Packaging and Packaging Waste Regulation (PPWR), which from 2030 will mandate a minimum recycled content in new packaging at a level of 10–35% and introduces a requirement for the full recyclability of all plastic packaging by 2035, the limitations of mechanical recycling methods represent a significant hurdle. While mechanical recycling remains highly effective for clear, well-sorted bottle streams, it struggles with more complex feedstocks such as thermoformed trays, colored or black PET, and PETG. Furthermore, chemical recycling is essential for managing polyester textile waste, a high stream that is virtually unaddressable via conventional mechanical methods. Meeting stringent legislative requirements and broadening the scope of recyclable feedstocks thus necessitates the implementation and scaling up of advanced chemical recycling technologies. An overview of key legal acts stimulating the development of the recyclate market in Europe is presented in Table 1.

## 3. PET Recycling Methods

### 3.1. Mechanical Recycling

Mechanical recycling is currently the dominant technology for PET waste management. It involves the physical reprocessing of waste, which inevitably impacts the chemical structure of the polymer chain through changes in molar mass and dispersity as a consequence of transesterification and hydrolysis [42]. The recyclate (rPET) obtained in this way can be reused in the production cycle in a strictly defined proportion that will not significantly reduce the end-use properties of the final product.

The key disadvantage of this method is the limited number of possible processing cycles. Each processing cycle results in gradual and irreversible thermomechanical degradation, leading to the loss of the material’s original physicochemical properties [43]. Consequently, the growing and legislatively enforced demand for rPET, combined with its progressive degradation, leads to a systematic decline in the quality of the secondary raw material. This deterioration is especially pronounced today due to the widening of collected feedstock sources, which increasingly include complex and harder-to-process fractions such as thermoformed trays, colored or opaque PET, and PETG [44].

It should be noted that the inherent polymer chain degradation associated with conventional mechanical recycling can be partially mitigated through Solid-State Polycondensation (SSP). This process effectively increases the intrinsic viscosity and molecular weight of the recycled PET, restoring its physicochemical properties and making closed-loop (bottle-to-bottle) applications feasible. Nevertheless, for more heavily degraded or complex waste streams, chemical recycling remains necessary.

### 3.2. Energy Recovery

PET is characterized by a high heating value (22–24 MJ/kg), which is comparable to that of hard coal and exceeds that of lignite [45]. This fact makes energy recovery an economically viable method of waste management.

Nevertheless, the high level of CO_2_ emissions and the irretrievable loss of a valuable raw material dictate that energy recovery is considered a last resort. Currently, the material stream directed to energy recovery consists mainly of highly contaminated waste, multi-material packaging (such as laminates), difficult-to-sort fractions like black plastics, and, additionally, plastics that have undergone such severe degradation due to repeated processing that it no longer meets the quality requirements for material recycling [43].

### 3.3. Chemical Recycling

Chemical recycling is currently the subject of intensive research, development, and implementation efforts. The essence of this process is the PET depolymerization reaction, which can follow two distinct technological pathways depending on the target application: full glycolysis, leading to the generation of a monomer stream (primarily BHET, which can be further converted to PTA and MEG) for conventional closed-loop recycling, or partial glycolysis, yielding a stream of functional oligomers and low-molar-mass glycolysates. While these oligomeric mixtures are predominantly tailored for open-loop conversion (e.g., into polyurethanes), they are also increasingly utilized in highly energy-efficient closed-loop approaches (such as the CuRe technology) for direct repolymerization back into virgin-grade PET [46,47,48,49,50,51,52,53]. These products can be used for the resynthesis of the virgin polymer (repolymerization) or for the production of new, derivative products (e.g., specialty polyols, resins, and adhesives) [54,55,56]. It is important to note the ongoing debate regarding the terminology of “upcycling” in the context of plastics. As explicitly advised in recent conceptual frameworks, diverting PET from closed-loop (PET-to-PET) recycling towards other polymer classes is scientifically debated, as true circularity favors maintaining the original polymer identity [57].

The fundamental advantage of chemical recycling methods is their high tolerance regarding the quality of the feedstock. Unlike mechanical methods, which are highly sensitive to contamination, chemical recycling allows for the processing of soiled waste, multilayer materials, and plastics severely degraded through multiple processing cycles [10,58].

Recent scientific and technological reports also indicate the possibility of conducting chemical recycling via a continuous reactive extrusion process. These studies have proven that solvent-free PET depolymerization into liquid polyols is achievable directly within the plasticizing unit of an extruder. The combination of high shear stress and an appropriate catalytic system effectively eliminates the phase separation problem, enabling continuous and fully scalable production [59]. Evaluating reactive extrusion under industrially relevant operating conditions highlights its significant kinetic and economic advantages over conventional batch reactors. The intense shear conditions (typically achieved at screw speeds of 100–300 rpm) drastically enhance mass transfer between the highly viscous PET melt and the depolymerizing agent. This intense mixing allows for near-complete conversion efficiencies (>95%) within remarkably short residence times of just 2 to 10 min. Depending on the extruder size, PET throughputs can easily scale from pilot lines (tens of kg/h) to full industrial capacities (several tons/h). From an economic standpoint, the energy consumption is substantially reduced—often by over 50—due to the continuous nature of the process and the elimination of massive solvent volume heating. The final product viscosity can be precisely tuned in-line by adjusting the glycol-to-PET ratio and temperature profile, yielding liquid polyols ready for downstream synthesis. Crucially, regarding storage stability, the architecture of modern extruders equipped with continuous vacuum venting zones enables the efficient, in-line evaporative removal of released MEG and by-products [59]. As will be discussed further, this active removal of MEG is a paramount mechanism for disrupting molecular symmetry and significantly prolonging the phase stability of the obtained glycolysates.

## 4. Chemical Fundamentals of Depolymerization Process

Unlike polymers with a purely carbon backbone (such as polyolefins or PVC), which are highly resistant to depolymerization, PET, as a heterochain polymer, exhibits high susceptibility to chemical recycling processes [60,61]. The presence of polar, regularly distributed ester bonds in the polymer chain enables controlled depolymerization at a relatively low energy input [62]. The strong polarization of the carbonyl group in the ester bond creates an electrophilic centre, providing an ideal site for a nucleophilic attack [63,64]. This attack leads to the formation of a high-energy tetrahedral intermediate; the activation barrier associated with its formation and subsequent decomposition governs the reaction rate.

This relatively low activation barrier for bond exchange reactions—especially when compared to the chemically inert C-C bonds in polyolefins—allows the depolymerization process to be carried out under relatively mild temperature conditions (180–220 °C) [65,66,67,68]. To further lower this activation barrier, current research trends focus on the application of novel, advanced catalytic systems: ionic liquids [61,62], nanometallic catalysts [69,70,71,72], deep eutectic solvents (DES) [70,73,74] and alternative energy sources (microwave irradiation [69,73], photoreforming [75,76], ultrasound [77,78]). In industrial practice, the process parameters are often constrained by physicochemical limitations resulting from the specific depolymerizing agent used (e.g., its boiling point) [68].

**Figure 8:**
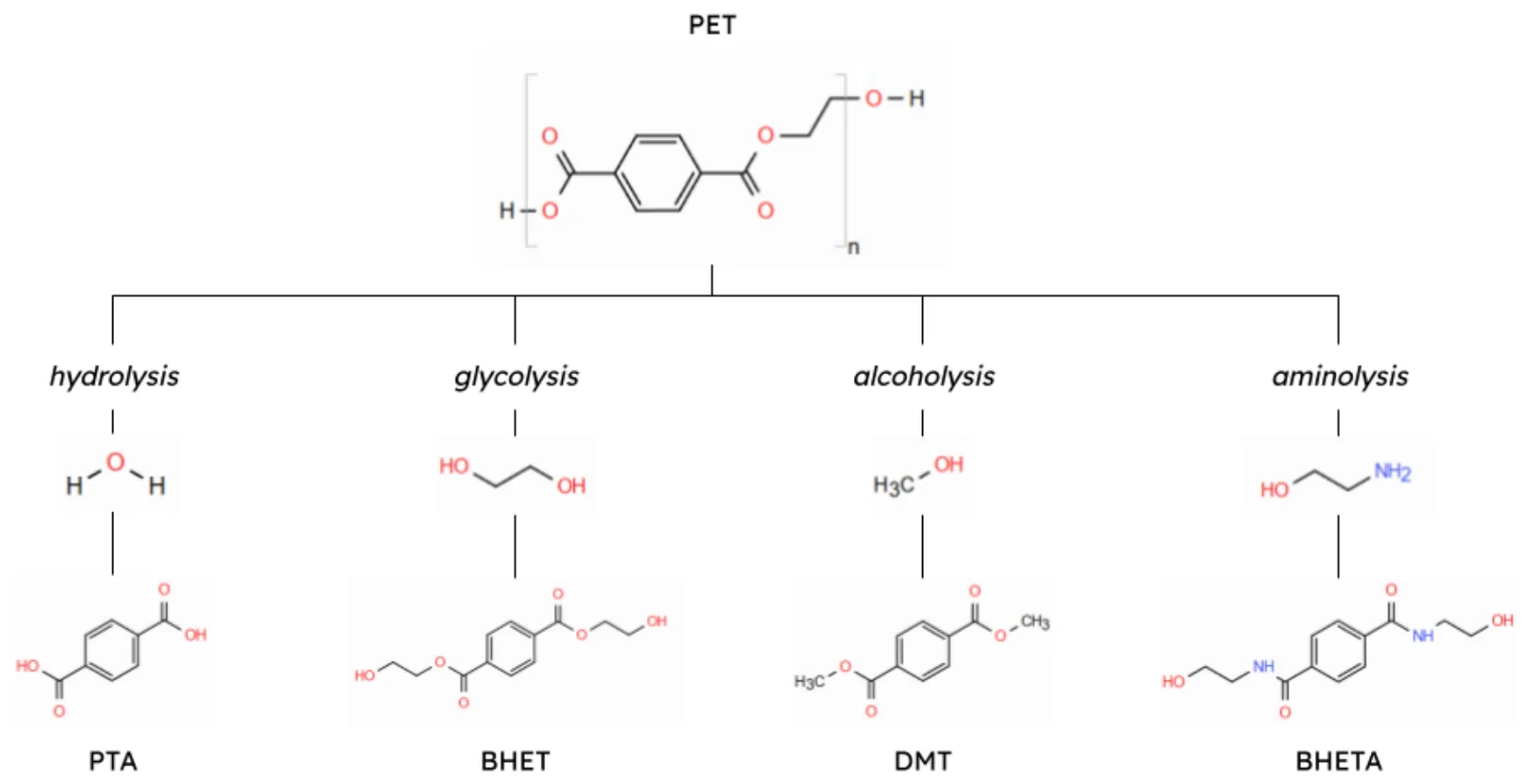
Main routes of PET chemical recycling.

Depending on the nucleophilic agent applied, several main depolymerization routes can be distinguished, which are summarized in Figure 8.

### 4.1. Aminolysis

This method utilizes primary amines (e.g., ethylamine, ethanolamine) to synthesize symmetrical diamides of terephthalic acid [79,80]. PET aminolysis products, such as bis(2-hydroxyethyl)terephthalamide (BHETA), are valuable intermediates in the synthesis of derivative polymer materials, such as curing agents, epoxy resins, and rigid polyurethane foams [81,82].

### 4.2. Methanolysis/Alcoholysis

This method was aimed at recovering DMT and MEG through the reaction of PET with methanol. Currently, it is losing industrial significance due to the challenges associated with managing highly toxic methanol and the necessity of utilizing expensive pressurized reactors [83]. While early industrial attempts faced challenges with toxic methanol management, modern mega-scale commercial projects (such as those operated and commissioned by Eastman) successfully utilize methanolysis for high-volume, closed-loop recycling back to virgin-quality rPET, boasting highly favorable Life Cycle Assessment (LCA) metrics [62]. Simultaneously, alternative alcoholysis trends explore alternative pathways, utilizing higher alcohols (C8, C17–C20) for the direct synthesis of specialty polyesters and PVC plasticizers [84,85].

### 4.3. Hydrolysis

This process leads directly to the recovery of PTA and MEG. Commercial hydrolysis processes, however, encounter significant technological barriers: alkaline hydrolysis generates large amounts of wastewater that is difficult to treat, acid hydrolysis requires the use of expensive acid-resistant steel alloys, and neutral hydrolysis demands extreme temperature and pressure conditions (using supercritical water) [47,52].

In view of the environmental, economic, and technological limitations, glycolysis remains the method with the highest implementation and market potential, thereby constituting the main subject of this review.

## 5. PET Glycolysis

### 5.1. Mechanism

Contemporary kinetic models define PET glycolysis as a complex, equilibrium-driven transesterification process conducted in an excess of glycol, initially occurring at the liquid–solid phase boundary [86,87,88,89].

In the first stage, the heterogeneous nature of the mixture means that the diffusion of glycol into the solid polymer phase initially limits the overall rate. To quantitatively distinguish between the kinetic regimes, reported activation energies must be evaluated. The activation energy (E_A_) for glycol diffusion is relatively low (typically 15–30 kJ/mol). Therefore, the initial phase is predominantly diffusion-controlled. However, as the polymer swells, cleaves, and dissolves, the system transitions into a homogeneous state. In this chemically controlled regime, the overall reaction rate is dictated by the nucleophilic attack and the subsequent formation of an unstable, high-energy tetrahedral intermediate [90].

The activation energy for this transesterification step is significantly higher, generally reported between 80 and 110 kJ/mol for uncatalyzed systems, which can be effectively reduced to 50–70 kJ/mol in the presence of efficient transesterification catalysts. Analyzing these parameters confirms that elevated temperatures and optimal mass transfer are essential to quickly overcome the initial diffusion barrier, shifting the process into the chemically controlled regime where catalyst efficacy becomes the dominant factor [63,67,89,91].

**Figure 9:**
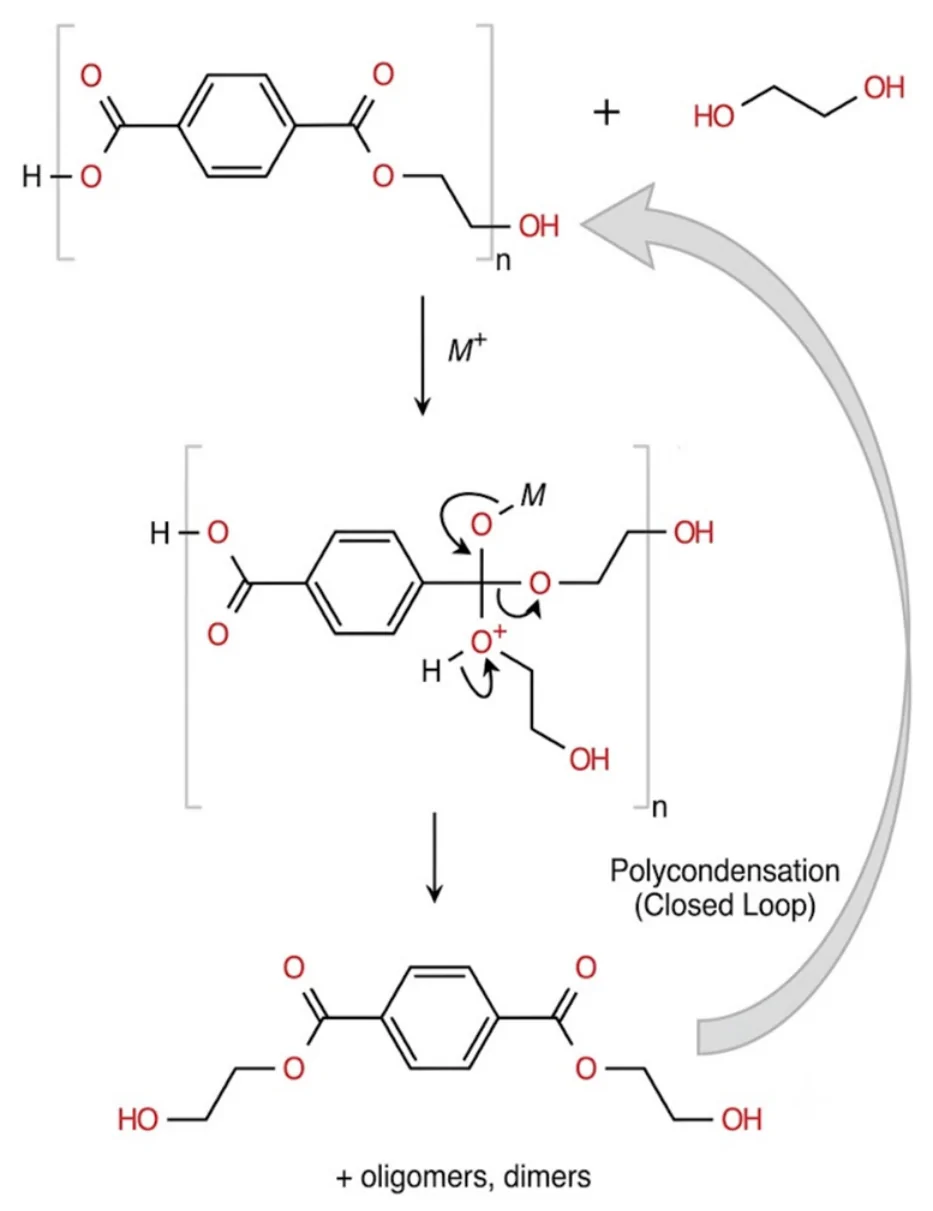
PET glycolysis mechanism.

Comparative studies indicate that larger glycol molecules containing ether bonds, e.g., diethylene glycol (DEG), due to a strong steric hindrance effect, generate significant spatial crowding in the transition state. This significantly impedes the effective approach of the glycol molecule to the electrophilic reaction centre [92]. As the reaction progresses, the system undergoes complete homogenization. The final composition of the reaction mixture, which is an equilibrium system of monomers (depending on the agent used, e.g., bis(2-hydroxyethyl) terephthalate (BHET) when using ethylene glycol), dimers, and higher oligomers, is strictly determined by the process parameters, in particular, the molar ratio of the glycol used to the polymer, as well as the reaction temperature and time [87,89,93]. The general mechanism of the PET glycolysis process is showed in Figure 9.

### 5.2. Process and Technological Parameters

A review of the literature highlights several key directions for optimizing glycolysis, depending on the variables adopted:

#### 5.2.1. Type of Glycol and Co-Solvents

The choice of the glycol type and co-solvents strongly depends on the targeted recycling pathway: whether the process aims at a return to PET via closed-loop recycling, where monoethylene glycol (MEG) is essentially the only option to regenerate the original polymer structure, or at open-loop conversion into functional polyols or other polymer classes. The reactivity of different glycols in the PET glycolysis process depends on the chain structure; for example, the presence of ether bonds in DEG can facilitate polymer swelling, despite the increased steric hindrance compared to MEG. Other glycols, such as 1,4-butanediol [92], propylene glycol, and neopentyl glycol [86], have also been successfully applied. It has been shown that glycols containing secondary hydroxyl groups exhibit lower reactivity than primary glycols [84]. Earlier studies have verified the possibility of using mixtures of glycols and complex oligoesters (e.g., products of the transesterification of dimethyl iso-phthalate with neopentyl or tetraethylene glycol) [94,95,96]. To improve environmental indicators, bio-based raw materials are being analyzed, including castor oil [97], rapeseed oil [98,99] and bio-MEG [94]. Promising results have been achieved by using gamma-valerolactone as a co-solvent, which allows for up to a five-fold acceleration of the reaction at a temperature of 215 °C [87].

#### 5.2.2. Catalysts

Transesterification catalysts play a crucial role in increasing the efficiency of PET glycolysis [100], enabling a reduction in the process temperature and shortening the reaction time [101]. On an industrial scale, heterogeneous catalysts demonstrate an advantage over homogeneous ones due to their easier separation from the reaction mixture and generally lower toxicity [102]. Classical salts and transition metal oxides (e.g., Sb_2_O_3_ [103], Zn(Ac)_2_) are currently being replaced by highly efficient alternatives: zinc-modified mesoporous zeolites [93], Pd-Cu/Al_2_O_3_ complexes [104] or polyoxometalates [105]. A significant performance leap has been reported for cyclic copper complexes (98% conversion at 160 °C) [90], ionic organocatalysts [106] and ionic liquids [107]. The application of in situ generated oxygen-vacancy-controlled Fe/ZnO nanosheets exhibits a 56% energetic benefit and a reduction in LCA indicators [64].

Recent studies devote considerable attention to deep eutectic solvents (DES) based on metal acetates (Zn, Co, Pb, Mn), which, acting simultaneously as a reaction medium and a catalyst, allow for 100% PET conversion [92,108]. The latest reports highlight a clear research gap regarding the need to remove catalysts after the glycolysis process [109].

Furthermore, while current literature predominantly evaluates catalyst performance based almost exclusively on overall PET conversion, this one-dimensional metric is insufficient for industrial scale-up. To enable meaningful visual and quantitative comparisons between various processes in the future, catalytic studies must broaden their evaluation criteria. It is highly recommended that researchers routinely report BHET selectivity, detailed oligomer mass distributions, and true turnover efficiencies (Turnover Number (TON) and Turnover Frequency (TOF)). Additionally, comprehensive evaluations of polymer purification efforts and the actual recyclability of the catalyst over multiple operational cycles must become standard practice to determine the true industrial viability of novel catalytic systems.

Optimizing the catalyst concentration is a critical parameter for controlling the kinetics of the glycolysis reaction. Zinc acetate (Zn(Ac)_2_) exerts a distinct influence on the degree of PET chain depolymerization. The precise dosing of the catalyst allows for controlling the efficiency of ester bond cleavage, which directly translates into the chemical structure (e.g., degree of hydroxylation and molecular weight distribution) of the obtained oligoester polyol [110].

#### 5.2.3. Waste PET Feedstock Quality

The glycolysis process occurs much faster in loosely packed amorphous regions than in crystalline ones, which results from their easier accessibility to diffusing glycol molecules and the catalyst [66]. Crystalline areas are more resistant to chemical attack, requiring higher activation energy, a higher temperature, or a longer time to effectively carry out depolymerization [111]. Due to the heterogeneous nature of the initial stage of glycolysis, the reaction rate depends on the degree of surface area developed that is in contact with the glycolyzing agent [67,87,91]. Proper preparation of the raw material prior to synthesis is equally important. In the presence of metallic catalysts (ZnO, Mn_2_O_3_, Zn(Ac)_2_), while high moisture levels in waste PET might not completely halt the initial conversion of the polymer, the yield of BHET formation and the process selectivity drop drastically as a result of competitive hydrolysis reactions [112].

To mitigate the high variability in glycolysis outcomes caused by feedstock inconsistencies, it is highly recommended that future studies adopt a standardized feedstock-characterization protocol. The magnitude of interconversion reactions, often mediated by agents such as trace water or metallic impurities, necessitates moving beyond simple generic descriptions of “post-consumer PET”. We propose that a comprehensive ‘feedstock passport’ should be routinely reported, encompassing the following baseline parameters:

- Crystallinity degree via Differential Scanning Calorimetry (DSC)**:** heavily influencing the initial glycol diffusion rate;
- Intrinsic viscosity: reflecting the initial polymer chain length and prior thermomechanical degradation history;
- Particle-size distribution (PSD) and surface area: dictating the solid-liquid mass transfer boundary;
- Moisture content: crucial for predicting competitive water-mediated hydrolysis during depolymerization;
- Residual-metal concentrations and colorants: which can inadvertently act as heterogeneous co-catalysts or catalyst-poisoning agents.

Implementing such a standardized characterization system would bridge the gap between varying experimental conditions, allowing for a much more reliable and rigorous comparison of catalytic efficiencies and kinetic parameters across the global literature.

#### 5.2.4. PET Concentration

To facilitate accurate comparisons across the rapidly growing body of literature, it is highly recommended that future studies standardize the reporting of reactant proportions using a dual-metric system: explicitly stating both the molar ratio of glycol to PET repeat units and the initial waste PET mass fraction (%) in the total reaction mixture. For instance, using a large excess of glycol (typically >70% glycol by mass, corresponding to a molar ratio often exceeding 4:1 to 6:1) significantly lowers the initial viscosity of the system, effectively reducing diffusion resistance at the phase boundary [87,89,91]. Under such conditions, the reaction equilibrium rapidly shifts towards the formation of pure monomers (BHET or DHET), which represents the primary objective for closed-loop repolymerization back into virgin-grade PET. However, for open-loop “PET-to-polyol” pathways, operating at such high solvent excess is highly counterproductive. For these open-loop applications, maximizing the waste PET feed is crucial not only to meaningfully improve the environmental indicators (e.g., lowering the carbon footprint) of the resulting functional polyols but also to comply with specific national legislative requirements and Green Public Procurement guidelines that mandate a minimum recycled content in construction materials (e.g., CAM Edilizia for PU/PIR foams in Italy) [32,62,113].

#### 5.2.5. Process Temperature

A constraint for the process conditions is the boiling point of the applied glycol, as summarized in Table 2.

Even when high-boiling glycols (DEG, TEG) are used, the threshold temperature of 197 °C (the boiling point of MEG, which is released in the transesterification reaction from PET chains) constitutes a critical point in the technology. Operating the process above this temperature results in the continuous distillation of MEG from the reaction mixture. According to Le Chatelier’s principle, the systematic removal of MEG from the system shifts the equilibrium towards further depolymerization, simultaneously preventing the secondary incorporation of the monomer into the oligomer chains. This fact is often overlooked in the literature, even though it significantly impacts the physicochemical parameters of the final product (a decrease in the hydroxyl number, an increase in viscosity) and its storage stability [101,106,107].

To effectively harness this distillative advantage without compromising the target polyol specifications, a rigorous practical operating window must be maintained. Industrially relevant conditions typically involve operating at temperatures of 200–220 °C under a gradually applied moderate vacuum (e.g., 200–500 mbar). This ensures the selective stripping of MEG while preventing the excessive loss of higher-boiling co-solvents (such as DEG). The extent of MEG removal must be carefully balanced—typically targeting the extraction of 30–50% of the theoretically released MEG. Exceeding this distillation threshold leads to a drastic increase in oligomer chain length and an exponential rise in viscosity, potentially rendering the polyol unprocessable. Consequently, the exact mass of the distilled MEG must be strictly monitored and integrated into the mass balance to precisely recalculate the final hydroxyl number. This continuous analytical correction is a critical requirement for establishing the correct isocyanate index during subsequent polyurethane formulations.

The kinetics of the glycolysis process can be controlled by manipulating the supplied thermal energy, as well as the type and amount of catalyst. It has been shown that the conversion achieved at 220 °C in a non-catalytic process can be fully replicated at just 190 °C through the addition of 500 ppm of tetra-n-butyl titanate [89].

#### 5.2.6. Energy Delivery Methods

The method of energy delivery significantly affects heat transfer efficiency and the overall rate of the glycolysis process. Conventional laboratory and industrial systems rely on convective heating. Although this is a well-established method, it is inextricably linked to slow heat transfer. The application of microwave irradiation has proven to be a breakthrough direction [114,115,116]. Unlike convective heating, microwaves interact directly with polar molecules throughout the entire volume of the mixture. It has been demonstrated that the use of a 400 W microwave field in a MEG environment with DES as a catalyst allows for achieving 100% PET conversion in just 5 to 9 min at a temperature of 180 °C [108]. However, implementing microwave technology on a production scale is associated with very high capital expenditures.

### 5.3. Industrial Scale-Up and Commercial Case Studies

While laboratory-scale research focuses primarily on kinetic optimization, alternative heating methods and novel catalyst development, the transition to the industrial scale introduces severe engineering challenges. These include mass transfer resistance in highly viscous melts, catalyst recovery and economic feasibility. Over recent years, several technologies have bridged this gap, bringing PET glycolysis to commercial or advanced pilot stages.

A prominent industrial case study is the technology developed by Ioniqua Technologies (Nederlands), which utilizes proprietary magnetic particle-supported ionic liquid catalysts. This approach allows for the processing of low-grade PET waste into high-purity BHET, elegantly solving the post-reaction catalyst separation issue through magnetic recovery [117,118]. Another notable industrial example is a technology developed by JEPLAN (Japan), which operates a commercial-scale facility employing conventional continuous glycolysis coupled with advanced purification columns to remove impurities, colorants and additives, enabling a closed-loop recycling material [119,120]. Furthermore, CuRe Technology operates a pilot loop focused on partial depolymerization. Rather than fully converting PET to BHET monomers, this process targets short-chain oligomers with a focus on separation and purification processes. This approach drastically reduces thermal energy demand and shortens reaction times, making it highly advantageous for direct integration into existing virgin polyester production lines [118,121].

These commercial implementations demonstrate that optimizing parameters such as the PET:glycol ratio—often restricted in industrial reactors to minimize the high energy costs associated with excess glycol distillation—is critical for making chemical glycolysis economically competitive with virgin polymer systems [62,118]. Moreover, this specific approach to running the reaction has a distinct target, namely full BHET conversion [122]. On an industrially integrated scale, heat integration can help recover part of the heating efforts. It is also important to note that in most Life Cycle Assessments, the energy demand for monomer purification (including MEG distillation) significantly outweighs the energy need of the depolymerization reaction itself [62].

To systematically evaluate these commercial implementations, a quantitative comparison of the leading industrial chemical recycling technologies is presented in Table 3. It should be noted that while exact economic parameters (e.g., operating costs and specific energy demand) remain proprietary commercial secrets, general Life Cycle Assessment (LCA) indicators confirm that maintaining optimized catalyst and glycol recovery systems is essential for achieving a favorable carbon footprint compared to virgin PET production.

### 5.4. Chemical Characteristics of PET Glycolysis Products

The products obtained as a result of rPET glycolysis constitute a complex chemical mixture, the composition of which is determined not only by the process parameters but also by the purity and origin of the waste feedstock.

Complete depolymerization in an excess of MEG (molar ratio > 4:1) leads to the formation of crystalline bis(2-hydroxyethyl) terephthalate (BHET). It is important to reiterate that this represents a strict closed-loop recycling target, fundamentally distinct from the open-loop polyol applications discussed later. In this regime, maximizing the yield and purity of crystallized BHET is not a phase-stability nuisance, but rather the core operational objective for large-scale industrial PET glycolysis operators, as it allows for direct repolymerization into virgin-grade PET [122].

The use of a glycol other than MEG results in a complex mixture of products. This is due to the fact that the MEG released from the polymer chain during depolymerization competitively participates in reversible transesterification reactions. Consequently, the reaction environment yields terephthalate esters corresponding to the active alcohol used, regenerated BHET, and mixed esters [89,95].

It has also been demonstrated that at a low glycol: PET molar ratio (often below 2:1), the solvent deficit prevents complete depolymerization, forcing the reaction to stop at the stage of dimers and higher oligomers. The mass distribution of the products can, in this case, be controlled by the temperature and time of glycolysis [93].

The glycolysate contains a range of substances that significantly affect subsequent processing stages and can alter the quality of the final products in open-loop applications (polyurethanes or resins). A detailed breakdown of the components of rPET glycolysate is presented in Table 4.

In order to remove color impurities (pigments, dyes), activated carbon can be used, which provides effective decolorization of the glycolysis products (whiteness increases from 60 before to 95 after decolorization) and a reduction of the metal content in the glycolysate (Zn content decreases from 200 to 2 mg/kg) [88]. Other standard purification stages most commonly include hot filtration (removal of insoluble polyolefins) and vacuum distillation to recover the excess free glycol and remove volatile by-products (e.g., 1,4-dioxane) [122,123]. However, implementing these additional chemical work-up and purification stages to address initial feedstock impurities significantly increases the overall energy demand and operational costs of the recycling process.

## 6. Stability of PET Glycolysis Products

The chemistry of the glycolysis process is well understood and widely described in the literature. Nevertheless, the phase stability of the obtained products, as liquid mixtures, during storage remains a key and unresolved technological challenge. Obtaining a clear, homogeneous glycolysate immediately after the reaction is commonly achievable on a laboratory scale, regardless of the composition and waste PET content. Maintaining this homogeneity during long-term storage is a critical parameter determining industrial applicability. This aspect is often overlooked in scientific publications, which mostly focus on transient properties, neglecting the phenomena of secondary crystallization progressing after cooling [100,124].

PET is a semi-crystalline polymer. Its ability to crystallize results from the presence of rigid aromatic rings in the main chain. The para-orientation of the structure (relative to the terephthalic acid segments) contributes to the linearity and rigidity of the bonds. Additionally, these rings, connected by short aliphatic segments, promote the formation of strong intermolecular π-π stacking interactions [114]. This phenomenon occurs as a result of the electrostatic attraction between the delocalized electron clouds of adjacent, parallel-aligned aromatic rings [125].

During glycolysis, long polymer chains undergo depolymerization into shorter oligomers. The obtained oligomers from an incomplete reaction, retaining rigid, linear fragments of the terephthalate structure, exhibit a natural tendency to reorganize into densely packed crystalline networks. This results in secondary crystallization, which is the primary cause of the progressive clouding of glycolysis products [6,125]. This phenomenon intensifies drastically with an increase in the initial loading of waste PET used for glycolysis. Above 30–40%, maintaining stability in the liquid phase becomes impossible when using basic glycolysis methods [115].

The empirically observed threshold of 30–40% waste PET loading represents a critical thermodynamic point of supersaturation for rigid aromatic segments within the liquid matrix [115]. Scientifically, this limit is governed by the interplay of several key parameters:

- Oligomer Molecular-Weight Distribution (MWD): At high PET concentrations (which inherently means a lower glycol excess), the reaction equilibrium shifts towards longer, incomplete depolymerization products (dimers, trimers) [93]. These longer chains contain multiple intact terephthalate sequences that strongly tend to associate via π-π stacking [114,125].
- Glycol Chemistry: The structural nature of the depolymerizing agent dictates the packing density. When using short, symmetrical glycols (like MEG), the resulting oligomers perfectly mimic the highly crystallizable native PET structure. Utilizing bulky, branched, or ether-containing glycols (such as DEG or DPG) can slightly push this threshold higher by increasing conformational entropy and inter-chain distance [126,127,128].
- Moisture Level and Catalyst Type: High moisture content in the unpredried PET feedstock induces competitive hydrolysis alongside glycolysis. This generates terminal carboxylic acid groups that form strong intermolecular hydrogen networks, accelerating phase separation [112]. Furthermore, unrecovered solid catalysts or precipitated metallic species act as highly active heterogeneous nucleating agents, drastically reducing the induction time for crystallization [109,125].
- Cooling History and Storage Temperature: The thermal history strictly dictates the glycolysate’s shelf-life. Slow cooling allows oligomer chains sufficient time and mobility to organize into crystalline lattices. While rapid thermal quenching might temporarily trap the system in a metastable amorphous state, storing the liquid at temperatures that provide sufficient segmental mobility inevitably triggers spontaneous secondary crystallization over time [125].

While such crystallization is not an issue for closed-loop recycling, these physical states render the material completely unsuitable for open-loop polyurethane production technology, due to the inability to pump them without implementing economically unjustified heating systems [129,130].

To prevent the instability of glycolysates and polyester polyols synthesized with a high initial share of waste PET, methods aimed at disrupting molecular symmetry are employed. A summary of the impact of selected modifiers, process parameters, and catalysts on the phase stability time (shelf-life) is presented in Table 5.

An analysis of the methodology of the conducted syntheses indicates that the significant extension of the phase stability of modified glycolysates (up to 160–180 days) results not only from the steric hindrance effects introduced by adipic acid and glycerol. A key technological measure is the simultaneous removal of condensate from the reaction system. Due to the difference in boiling points, alongside the water from polycondensation, the MEG released during the transesterification process is distilled out of the reactor [115]. The active distillative removal of the highly symmetrical MEG—originating from the ester segments of the original polymer chain—drastically reduces the thermodynamic ability of the system to undergo secondary crystallization, as it ensures its permanent replacement with less symmetrical glycols within the oligomer backbone.

Nevertheless, rigorous quantitative mass balances and chromatographic analyses of the distillates are still lacking in the available literature. The lack of precise data determining the degree of conversion and the loss of MEG from the system precludes accurately correlating the final viscosity with the actual chemical composition of the stable polyol and establishing a reliable mass balance and recycled content calculation.

A frequently described practice is replacing MEG with longer ether glycols, such as DEG, TEG, and higher analogues (poly(ethylene glycol)) [114]. The C–O–C ether bond present in them is characterized by a significantly lower rotational barrier than the C–C bond present in the aliphatic segments of PET oligomers, effectively acting as a ‘swivel’. Simultaneously, the greater length of the DEG molecule (relative to the MEG present in the PET chain) increases the inter-chain distance between the aromatic rings, which exponentially weakens the strength of intermolecular interactions (in accordance with the Lennard-Jones potential) [126,127]. Substituting DEG for MEG in the oligomer chains significantly increases the conformational entropy of the oligomer, effectively hindering the formation of rigid crystalline structures [128].

Another effective method is introducing segments that generate steric hindrance into the structure of the glycolysate or polyol. For this purpose, branched-chain glycols or those containing methyl substituents are used (1,2-propanediol, glycerol, 2-methyl-1,3-propanediol, 3-methyl-1,5-pentanediol). The presence of side groups disrupts chain regularity, effectively hindering the association of aromatic rings and lowering the crystallization temperature [86,109,128].

An analogous symmetry-disrupting effect can be achieved through the addition of aliphatic acids (adipic acid), which dilute the packing of aromatic groups. Alternatively, phthalic anhydride is incorporated into the chain. As an ortho-phthalic acid derivative (1,2-substitution), it diametrically alters the chain geometry in contrast to linear terephthalate segments (1,4-substitution), permanently lowering the system’s ability for close packing [109,131].

## 7. Industrial Applications of PET Glycolysis Products

**Figure 10:**
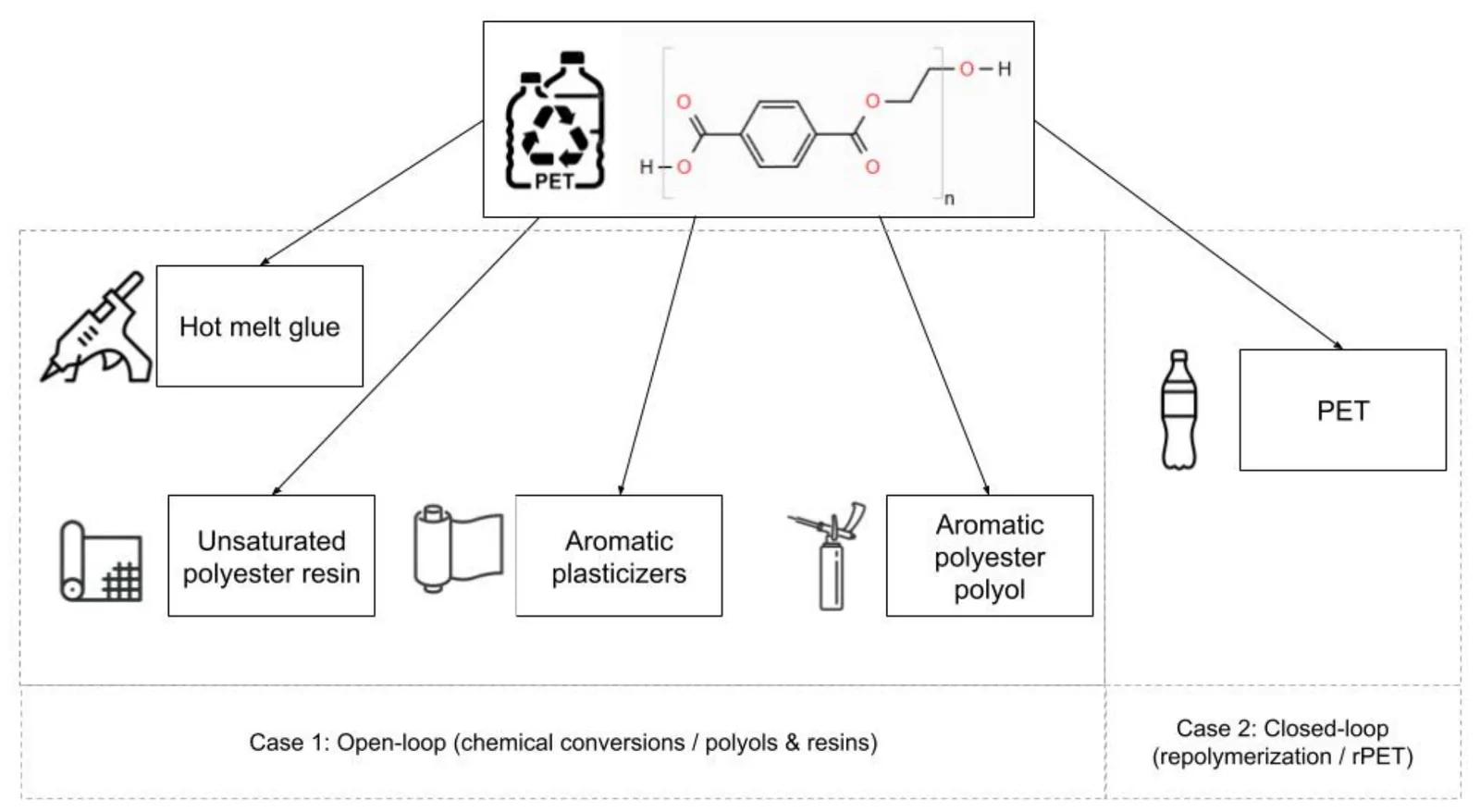
Main industrial applications of PET glycolysis products.

The primary functionality of PET glycolysis products is provided by their at least bifunctional nature (the presence of a minimum of two terminal hydroxyl groups). This determines their potential application in polycondensation processes with carboxylic acids or their anhydrides, enabling the synthesis of a wide range of polymeric materials. The depolymerization products of waste PET constitute a valuable base substrate for resins, adhesives, and polyurethanes, among others, as illustrated in Figure 10. However, it must be acknowledged that applications such as specialty polyols and resins are, volume-wise, a “niche” compared to the massive global PET packaging stream. Therefore, chemical transformation into alternative polymer classes should not be viewed as the ultimate end-of-waste solution for the entire bulk of PET waste—a role fulfilled by mechanical recycling, aided by Solid-State Polycondensation (SSP) and PET-to-PET depolymerization. Instead, these processes should be motivated as an excellent opportunity to access an attractive, non-fossil starting material for specific specialty chemical sectors.

### 7.1. Closed-Loop Repolymerization (PET-to-PET)

As emphasized throughout this review, the highest-volume target for PET glycolysis is closed-loop recycling. Complete depolymerization into highly purified monomers (primarily BHET) allows for direct repolymerization into virgin-grade PET. This pathway is crucial for achieving true circularity in the massive global packaging and textile sectors, fulfilling the ultimate end-of-waste criteria on a macro scale by permanently maintaining the original polymer’s identity.

### 7.2. Unsaturated Polyester Resins

In the polycondensation process of glycolysates, unsaturated carboxylic acids or their anhydrides (fumaric acid, maleic anhydride) are used. The resulting unsaturated polyester is dissolved in styrene to achieve the appropriate viscosity and concentration of unsaturated double bonds, which are capable of undergoing free-radical polymerization during the resin curing process [132]. Current research trends aim to eliminate toxic and volatile styrene, successfully utilizing bio-based raw materials such as bio-itaconic acid. PET-based unsaturated polyester resins are primarily used in the production of fiberglass composites and laminates.

### 7.3. Hot-Melt Adhesives

Glycolysis products constitute an excellent base for the synthesis of reactive hot-melt adhesives. The literature describes, among others, a synthesis using a propylene glycolysate (glycol:PET molar ratio of 2:1; catalyst: titanium(IV) iso-propoxide; temperature 180 °C). Following modification with, for instance, polyethylene glycol, the obtained glycolysate is reacted with an isocyanate (methylene diphenyl isocyanate (MDI)) to form a prepolymer. Its final molecular weight and adhesive properties can be precisely adjusted by adding a chain extender, such as 1,4-butanediol [94].

### 7.4. Plasticizers

Due to growing legal restrictions regarding the toxicity of classic orthophthalate plasticizers, waste PET depolymerization products represent an attractive raw material for the synthesis of safe alternatives. The reaction of PET oligomers (or pure PTA/DMT recovered from glycolysis) with higher aliphatic alcohols, such as 2-ethylhexanol, allows for the efficient production of dioctyl terephthalate (DOTP)—one of the most important plasticizers in the PVC processing industry [84,85]. Notably, unlike in polyurethane applications, the phase instability of the glycolysate is irrelevant here, as the subsequent high-temperature transesterification readily melts and converts any crystallized intermediates.

### 7.5. Polyester Polyols

Polyurethanes are polymers formed through the polyaddition reaction of diisocyanates with polyols containing available hydroxyl groups. Polyols form the structural backbone of polyurethane, directly determining its final physicochemical parameters. The use of glycolysates for the production of aromatic polyester polyols is highly justified both chemically and economically, while also being environmentally attractive [133]. However, as established in the introduction to this section, while the polyurethane market cannot absorb the massive global volumes of PET waste—reaffirming closed-loop repolymerization as the primary macroeconomic target—it remains a highly valuable, value-added niche for creating specialty non-fossil construction materials.

Due to the presence of aromatic rings, these polyols are particularly desirable in rigid insulation foams (PIR/PUR) and coatings. They provide high thermal stability, enhanced flame resistance, and high compressive strength [134,135]. The application of PET in the production of polyester polyols offers distinct advantages over phthalic anhydride-based polyols. The most significant benefits include improved fire resistance, better thermal stability, and a higher Limiting Oxygen Index (LOI) [131]. Polyurethanes based on waste PET-based glycolysates can also be used as binders for oxidizing agents and fuel in rocket engines [136].

A technological aspect affecting the scaling of the polyol polycondensation process is the removal of water from the system. Water, being a by-product, is collected as condensate, which is essential to shift the reaction equilibrium (according to Le Chatelier’s principle) and ensure the complete conversion of carboxylic groups. However, along with water molecules, glycol vapours are entrained, leading to material losses. This requires the adaptation of the reaction apparatus through the selection of appropriately designed distillation columns, minimizing raw material losses that are both economically and environmentally unfavourable [137].

### 7.6. Advanced Functional Materials

Alongside chemical depolymerization, waste PET can be directly physically transformed into high-value functional products, such as multi-functional core/sheath bicomponent nonwovens for thermal insulation [138]. However, comparing these direct physical pathways to chemical recycling reveals that glycolysis uniquely unlocks the synthesis of entirely new macromolecular architectures. Beyond standard polyurethanes, glycolysis intermediates (such as BHET and oligoesters) are increasingly “upcycled” into advanced materials that are impossible to obtain directly from the rPET melt [139]. It must be emphasized that due to a clear mismatch of scales, these applications do not constitute a macro-scale end-of-life (EoL) solution for the bulk PET waste stream; rather, they represent potentially advantageous pathways for utilizing glycolysates as raw materials for new, high-value “niche” applications. Recent commercial and research developments include UV-curable resins for stereolithography 3D printing [140], dynamic covalent vitrimers [56], and self-healing anticorrosive smart coatings [141].

The technology for synthesizing polyester polyols based on rPET glycolysates has currently reached full market maturity—Technology Readiness Level (TRL) 9. This is confirmed by the successful commercialization of these products on the European market by leading manufacturers in the polyurethane industry, including Purinova (Poland), LERG (Poland), PCC Rokita (Poland), Neo Group (Lithuania), Stepan (Germany), Synthesia (Spain), and COIM (Italy).

## 8. Environmental Analysis

Despite the widespread positioning of chemical recycling as the foundation of the circular economy, LCA indicators reveal the shortcomings of processes conducted at low waste PET concentrations. The literature is dominated by the tendency to conduct glycolysis using a significant excess of the glycolyzing agent (waste PET loading at the level of 10–30%), which allows for easily circumventing technological problems related to high viscosity and the secondary crystallization of products purely through a physical dilution effect.

From a sustainable development perspective, however, such an approach is highly debatable. In products with a low rPET concentration, the dominant portion of the reaction mass (70–90%) consists of raw materials (glycols) of petrochemical origin, primarily MEG and DEG. These compounds are characterized by a high carbon footprint (typically in the range of 1.5–2.5 kg CO_2_ eq/kg) [62,113]. Designing an “eco-friendly” polyol whose feed is based almost 80% on petroleum-derived raw materials barely reduces fossil fuel consumption compared to the synthesis of conventional polyether or polyester polyols based on phthalic anhydride or terephthalic acid [113].

The use of a large excess of the glycolyzing agent drastically deteriorates the energy balance. Heating and maintaining the reactor contents at a temperature of 190–220 °C, the volume of which is largely filled by the solvent (excess glycol), requires high amounts of energy for the inert reactor loading. In LCA analyses for such processes, the emission “hot-spot” ceases to be the waste itself and instead becomes the energy consumed to maintain the temperature of the depolymerization process for a small fraction of rPET [62,113].

For the chemical recycling of PET via the glycolysis process to be environmentally justified and bring a real reduction in LCA environmental indicators (including, among others, the Global Warming Potential—GWP), it is necessary to reverse the mass proportions. It has been proven that replacing petrochemical raw materials brings distinct environmental benefits (a significant decrease in GWP) only in formulations where the recyclate share reaches or exceeds the level of 40–50% [113].

Striving to conduct glycolysis at high waste PET concentrations is no longer merely a process optimization—for open-loop polyol production, it becomes a strict pro-environmental requirement. In contrast, closed-loop PET-to-PET recycling achieves highly favorable environmental metrics regardless of the initial feed ratio through efficient MEG recovery and full conversion to BHET [62]. Unfortunately, entering this technological regime for open-loop applications immediately highlights the unresolved problem of secondary crystallization and phase instability of glycolysates, making this area a critical and simultaneously the most deficient research gap in the current scientific literature regarding glycolysates intended for later secondary use as polyols.

## 9. Research Gap and the Role of Distillation

An analysis of the available scientific publications from recent years reveals a significant research gap in the approach to waste PET depolymerization and the synthesis of waste PET-based polyols.

The vast majority of studies focus on investigating the depolymerization rate and process yield, determined primarily by the obtained amounts of monomer (BHET), completely neglecting the actual composition of the reaction mixture [142,143]. The issue of long-term physical stability (shelf-life) is systematically overlooked, despite its crucial impact on the viability of the industrial application of the obtained products [114]. Most literature reports operate on conservative waste PET loadings in the range of 10–30%. Attempts to conduct research at waste PET concentrations exceeding 30–40% are rare, and the products obtained in them are often disqualified due to rapid crystallization [144,145].

The analysed studies rarely address the problem of controlling the reaction equilibrium through the distillative removal of MEG released during the process. Single reports have been noted in the literature where the glycolysis process was coupled with the distillative removal of MEG. However, these studies were aimed solely at improving the physicochemical parameters of the final polyurethane produced from the obtained glycolysis products. The impact of the conducted process on product stability and crystallization kinetics was completely omitted. Distillation of MEG from the reaction system is also described as a process required to remove the excess MEG used to obtain BHET [123].

The standard “one-pot” approach in a closed system results in the retention of the MEG released from the polymer chain in the mixture [142,146]. Despite the use of modifiers, the system always tends toward thermodynamic equilibrium [87,147]. Due to its smaller molecular size and higher mobility, primary MEG is a highly reactive competitor for DEG in the process. It is reincorporated into the oligomer chains, restoring symmetrical, strongly crystallizing BHET segments [6,148]. While this restoration is highly desired if the target is closed-loop repolymerization into rPET, it is highly problematic when the objective is the open-loop synthesis of phase-stable polyols.

There is a clear contradiction between the global drive to maximize the share of recycled raw materials (more than 40%) and the current state of the art. To overcome this barrier, a change in the technological process regime is required, aimed at the permanent removal of low-molecular-weight depolymerization products (mainly MEG) from the system. Such an approach can allow for precise control of the reaction equilibrium and the production of oligomers with a structure less susceptible to crystallization. Such an approach can allow for precise control of the reaction equilibrium and the production of oligomers with a structure less susceptible to crystallization.

Furthermore, while current literature often attributes enhanced stability to either distillative MEG removal or the addition of chemical modifiers (e.g., glycerol, adipic acid), these variables are frequently convoluted in published formulations. A critical direction for future optimization research is the application of rigorous factorial Design of Experiments (DoE). Such methodological approaches are urgently needed to critically deconvolute the individual and synergistic contributions of chemical modifiers, catalytic systems, and reactor configurations (closed reflux setups versus active distillative MEG removal) when evaluated at equivalent reaction conversions.

## 10. Conclusions and Future Perspectives

Current challenges in the field of PET chemical recycling mainly focus on the scalability and reproducibility of processes, the development of highly efficient catalytic systems, and the optimization of purification techniques for the final glycolysis products [109,149].

The conducted analysis of recent literature indicates a significant research gap regarding glycolysis processes at high PET concentrations. As demonstrated, over 80% of experiments were conducted using a conservative feedstock, typically not exceeding 35–40% relative to the mass of the glycolyzing agent. These studies, with few exceptions, are aimed at achieving the highest possible yield, usually measured as the percentage of PET conversion to BHET. The few publications attempting depolymerization at higher PET concentrations (over 40%) systematically disqualify the obtained products due to their high viscosity, phase instability, or tendency to crystallize.

The steadily growing market demand and legislative requirements (SUP, PPWR) are directing the chemical industry towards developing production technologies with increased shares of recyclates in final products (including polyester polyols and polyurethanes). Thermomechanically reprocessed plastics, which are no longer suitable for mechanical recycling as a result of progressive degradation, constitute an excellent feedstock for the synthesis of glycolysates and polyols, where the raw material requirements are significantly lower. It is essential to make further progress toward the ability to obtain stable PET chemical recycling products with an increased recyclate content.

The conducted review defines two critical areas for future research and development (R&D) efforts:

### 10.1. Aging Studies

There is a clear publication deficit regarding aging studies of the obtained glycolysates. The current reliance on semi-visual terms (e.g., cloudy, pasty, solid, crystallized) and undefined shelf-life durations lacks normative foundations. Future research must shift towards developing rigorous, accelerated-aging protocols. Such a diagnostic building block should encompass a defined cooling history during production and controlled storage temperatures. Characterization must integrate time-resolved turbidity measurements, comprehensive rheological profiling, and polarized optical microscopy to evaluate crystal particle-size distributions. Alongside Differential Scanning Calorimetry (DSC) for phase transition analysis, periodic hydroxyl-number determinations are essential to monitor progressing secondary reactions. Developing and implementing this multi-instrumental protocol would ultimately lead to predictive kinetic models for glycolysis products stability, standardizing quality assurance frameworks that are currently missing in the industrial-scale formulation of phase-stable polyols.

### 10.2. Distillative Disruption of Crystallinity

The addition of physical chain disruptors is the simplest and most widely studied method; however, on an industrial scale, every additive impacts the final price of the product. A consequence of incorporating new components is the risk of failing to meet the structural requirements for the subsequent application of the obtained glycolysates and polyols (including in polyurethanes). The elimination of free MEG from the mixture and from the PET chains constitutes a highly promising solution to this problem.

## Figures and Tables

**Table 1:** Overview of key legal acts and fiscal instruments enforcing the use of recycled plastics.

Legal Act	Scope/Country	Required Recyclate Content or Imposed Sanction (Fiscal Instrument)	Refs.
ELV (End-of-Life Vehicles)	European Union	Min. 15% (increasing to 20%) of plastics in new vehicles must come from recycling, of which min. 20% must be derived from end-of-life vehicles.	[28]
PPWR (Packaging and Packaging Waste)	European Union	10% to 35% depending on packaging type (e.g., 30% for contact-sensitive packaging).	[29]
SUP (Single-Use Plastics)	European Union	Min. 25% rPET in bottles by 2025 and 30% by 2030.	[21]
Act on entrepreneurs’ obligations (Plastic Act)	Poland	Sanction (product fee, e.g., 1 PLN/kg) for failing to meet the requirement of 25% rPET in bottles (2025) and 30% (2030).	[30]
VerpackG (Packaging Act/Eco-modulation)	Germany	Enforcement of financial premiums (discounts in EPR fees) for the use of recyclates and design for recycling (§ 21)	[31]
CAM Edilizia (Minimum Environmental Criteria)—Green Public Procurement (GPP) instrument	Italy	2% to 10% certified recycled material content in rigid insulation foams (PUR/PIR).	[32]
Plastic Packaging Tax (PPT)	United Kingdom	30%; below this value, a tax of 217.85 GBP per ton of product applies.	[33,34]
Plastic Tax (Impuesto especial sobre los envases de plástico)	Spain	Tax of 0.45 EUR/kg. The tax exemption amount is directly proportional to the % of certified recyclate content.	[35]
Loi AGEC & RE2020	France	Carbon footprint limits for buildings and EPR eco-modulation favoring the use of recyclates, e.g., in construction and furniture.	[36,37]
Taxa sobre Embalagens	Portugal	Tax of 0.30 EUR on each single-use plastic packaging.	[38]
Environmental Fund Contribution (AFM)	Romania	Penalty fee of 2 RON/kg (approx. 0.40 EUR/kg) for failure to meet national plastic recycling targets.	[39]
EPR Eco-modulation (Afvalfonds/Fost Plus)	Netherlands Belgium	Stringent fees in EPR systems for producers placing virgin PET on the market, with significant relief (reduced tariffs) for rPET.	[40,41]

**Table 2:** Characteristics of glycols most commonly used in the PET glycolysis process.

Name	Structural Formula	Molar Mass [g/mol]	Boiling Point [°C]
ethane-1,2-diol (monoethylene glycol)	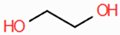	62.07	197
2,2′-oxydiethanol (diethylene glycol)	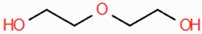	106.12	245
2- [2-(2-hydroxyethoxy)ethoxy]ethan-1-ol (triethylene glycol)	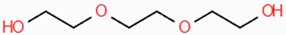	150.17	285
propane-1,2,3-triol (glycerol)	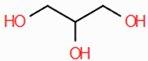	92.09	290
2,2-dimethylpropane-1,3-diol (neopentyl glycol)	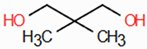	104.15	208
propane-1,2-diol (monopropylene glycol)	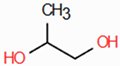	76.09	188
4-oxa-2,6-heptanediol (dipropylene glycol)	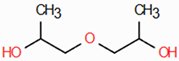	134.17	230

**Table 3:** Comparative overview of selected industrial-scale PET chemical recycling technologies.

Company/Technology	TRL	Estimated Processing Capacity	Feedstock Tolerance	Product Purity/Intermediate	Key Process Features (Catalyst & Recovery)
Ioniqa (glycolysis)	8–9	~10 kt/year (demonstration)	Low-grade, colored PET packaging	Virgin-grade BHET	Magnetic ionic liquid catalyst; highly efficient physical separation and catalyst recovery
JEPLAN (glycolysis)	9	~22 kg/year (commercial)	PET bottles, polyester textiles	High-purity BHET	Continuous glycolysis couples with advanced purification columns for colorants/additives
CuRe (partial glycolysis)	7–8	Pilot scale	Opaque PET, colored, textiles	Purified short-chain oligomers	Partial depolymerization significantly lowers thermal energy demand; in-line filtration
Eastman (methanolysis)	9	>100 kt/year (mega-scale)	Hard-to-recycle, mixed textiles	Virgin-grade DMT and MEG	Highly favorable LCA metrics; advanced methanol recovery

**Table 4:** Chemical composition and potential contaminants of PET glycolysis products.

Category	Component	Origin/Formation Mechanism	Impact on Product or Process
Main products	BHET/DHET	Main depolymerization products	Substrate for the synthesis of PET (repolymerization) or polyols
	PET oligomers	Products of incomplete depolymerization (dimers, trimers)	Increase viscosity, may precipitate in polyols. Conversely, in targeted partial depolymerization (e.g., CuRe approach), they lower melt viscosity sufficiently to enable filtration and purification, offering a highly energy-efficient route prior to direct repolymerization.
	Glycols	Residue of the excess depolymerizing agent	Affects the hydroxyl number, viscosity and vapor pressure
By-products	1,4-dioxane	Cyclization product of glycol (especially DEG or under acid catalysis)	Toxic, volatile by-product; requires distillation
	Acetaldehyde	Thermal degradation of the PET chain	Causes an unpleasant odour and yellowing of the product
	Ether glycols	In situ formation of DEG molecules from MEG	Reduce the thermal resistance and barrier properties of PET
Impurities (from rPET feedstock)	PVC and decomposition products	Caps, labels	Deactivation of basic catalysts, requiring corrosion-resistant equipment or neutralization
	Polyolefins (PP/PE)	Caps, labels	Solid/non-polar phase; form a suspension, necessitating energy-intensive hot filtration
	Pigments and dyes	Colored bottles (blue, green, black)	Deterioration of optical parameters, requiring additional decolorization work-up that increases operational costs
	Metals and catalysts	Residues of Sb, Ge, Ti from primary PET synthesis	May uncontrollably catalyse further reactions (oxidation)

**Table 5:** The impact of process parameters and modifiers on the phase stability of rPET glycolysates.

Base Glycol and Modifiers	rPET Concentration	Process Conditions and Catalyst	Observed Stability Effect	Ref.
DEG (no additives)	30–40%	190–220 °C, Zn(Ac)_2_	28 days (product melting point 42 °C)	[107]
MEG (no additives)	23.5%	190–250 °C, 4 h, Zn(Ac)_2_	White, crystalline paste	[107]
DEG 1–12% Glycerol, 16% Adipic Acid	41–48%	Dibutyltin dilaureate—2 drops	120–140 days	[107]
DEG 1% Glycerol, 16% Adipic Acid	40–41%	Dibutyltin dilaureate (15 ppm) Dibutyltin dilaureate (30 ppm)	160–180 days 120–140 days	[107]
DEG	40–41%	Titanium 2-ethylhexanoate (15 ppm) Titanium 2-ethylhexanoate (30 ppm)	60–80 days 60–80 days	[107]
DEG	40–41%	Tetrabutyl titanate (15 ppm) Tetrabutyl titanate (30 ppm)	20–30 days 20–30 days	[107]
Rapeseed Oil (RO)		Zn(Ac)_2_	2 months	[117]
MEG	16–40%	Microwaves 400 W, 240 °C Zn(Ac)_2_	White paste at 70 °C	[103]
MEG	16–40%	Microwaves 400 W, 240 °C Sb_2_O_3_	White paste at 70 °C	[103]

## Data Availability

No new data were created or analyzed in this study. Data sharing is not applicable to this article.

## Abbreviations

The following abbreviations are used in this manuscript:

PET	Poly(ethylene terephthalate)
rPET	recycled poly(ethylene terephthalate)
MEG	Monoethylene glycol
PTA	Purified terephthalic acid
DMT	Dimethyl terephahalate
PVC	Poly(vinyl chloride)
DES	Deep Eutectic Solvents
BHETA	bis(2-hydroxyethyl)terephthalamide
BHET	bis(2-hydroxyethyl) terephthalate
DEG	Diethylene glycol
TEG	Triethylene glycol
LCA	Life Cycle Assessment
